# Developing a Translational Team Training Program using the Wisconsin Interventions in Team Science Framework

**DOI:** 10.1017/cts.2023.649

**Published:** 2023-10-19

**Authors:** Whitney A. Sweeney, Patrick W. Kelly, Allan R. Brasier, Betsy Rolland

**Affiliations:** 1 Institute for Clinical and Translational Research, School of Medicine and Public Health, University of Wisconsin-Madison, Madison, WI, USA; 2 Carbone Cancer Center, School of Medicine and Public Health, University of Wisconsin-Madison, Madison, WI, USA

**Keywords:** team science training, translational teams, collaborative research, evaluation, evidence based

## Abstract

The Clinical and Translational Science Awards (CTSA) Program supports a national network of medical research institutions working to improve the translational process. High-performing translational teams (TTs) are critical for advancing evidence-based approaches that improve human health. When focused on content-appropriate knowledge, skills, and attitudes, targeted training results in the substantial internalization of training content, producing new skills that can be applied to improve team outputs, outcomes, and benefits. More rigorous approaches to develop, test, and evaluate interventions are needed, and we used the Wisconsin Interventions in Team Science framework as a model to systematize our efforts. We designed, built, and tested a five-session TT Training Program for translational researchers. The 90-minute sessions were pilot-tested with 47 postdoctoral fellows and evaluated through a structured evaluation plan. Ninety-five percent of post-session survey respondents indicated that the content and skills provided would make them more effective collaborators, and one hundred percent would recommend the sessions to colleagues. Respondents’ scores increased from pretest to posttest for most learning outcomes. Refinements from participant feedback are described. This work provides a foundation for the continued evolution of evidence-based training programs in the CTSA environment.

The Clinical and Translational Science Awards (CTSA) Program supports a national network of medical research institutions working to improve the translational process. High-performing translational teams (TTs) are critical for advancing evidence-based approaches that improve human health [1,2]. Translational researchers need skills to transcend beyond an individual discipline [3], but generally receive minimal training to establish high-functioning teams [4,5]. Poorly functioning teams are costly in terms of squandered effort and resources [6]. Thus, developing effective targeted training may be of value for TTs and the individual translational researchers who make up these teams [4]. When focused on content-appropriate knowledge, skills, and attitudes (KSAs), such training results in the substantial transfer of training, producing newly acquired skills that can be readily applied to improve team processes (e.g., enhanced research collaborations), outputs (e.g., increased publications and citations), outcomes (e.g., new therapies or interventions), and health benefits (e.g., polices or clinical guidelines) [1,7,8].

Over the last ten years, NIH has transitioned to funding more complex and inter-institutional grants. The number of multi-PI R01-equivalent grants has more than doubled and the number of data coordinating centers (U24s) funded has more than quadrupled since 2013 [9]. With this increase in project complexity, major funding organizations require formalized support for team activity led by CTSA programs [5]. A review of the CTSA websites shows that the training offered at each hub varies significantly regarding presentation mode, training duration, and, most importantly, the KSAs covered. The essential KSAs for supporting high-performing TTs have yet to be fully defined, implemented, and evaluated. [5] Thus, the evidence base supporting the impact of team science training for TTs is limited [8]. Articulating the KSAs and building the evidence base for effective team science training will make it easier for research institutes like CTSA hubs to strengthen the collaborative abilities of translational researchers and enhance their ability to impact human health [5,8].

The Team Science Core at the University of Wisconsin-Madison (UW Madison) Institute for Clinical and Translational Research (ICTR) is committed to using rigorous approaches to develop, test, and evaluate team science interventions that can ultimately be shared with other CTSAs. To systematize our efforts, members of the ICTR Team Science Core previously developed the Wisconsin Interventions in Team Science (WITS) framework (see Fig. 1) to guide the iterative process of translating team science strategies into evidence-based interventions. The WITS framework has four phases: (1) Discover (the problem space), (2) Design, build, and test (the prototype), (3) Conduct a pragmatic trial, and (4) Disseminate and implement the intervention [8]. This paper describes the first two phases of the WITS framework as applied to developing the TT Training Program and the initial pilot test conducted with postdoctoral fellows. The lessons learned from this first iteration led to modifications of the evaluation methods, a refocus of content, and the addition of techniques to engage audience members more fully. These changes were integrated into the next iteration of the training program with a future goal of expanding our work toward a broader dissemination (WITS phases 3 and 4).

**Figure 1.**
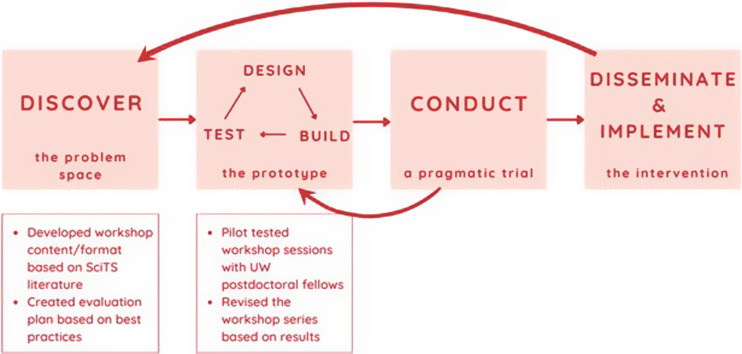
Translational Team (TT) Training Program Application of the Wisconsin Interventions in Team Science (WITS) Framework. Shown in the top portion of this figure are the four phases of the WITS framework. The bottom portion shows the steps taken for phases one and two of the WITS framework during the development of the TT Training Program and the associated evaluation plan. SciTS = Science of Team Science; UW = University of Wisconsin-Madison.

## Discover (the Problem Space)

The goals of the discover phase are to uncover the challenges that translational researchers face, understand the context in which they operate, and identify strategies to meet TT needs. Turning to the Science of Team Science (SciTS) literature is an important first step. The SciTS field not only describes key influences on science team process, culture, and leadership[8] but also specifically addresses TTs' unique needs [1,3,10,11]. As Director of ICTR’s Team Science Core, Dr. Betsy Rolland spearheaded the discover phase for the development of the TT Training Program. Using information derived from her design and implementation of TT interventions like Collaboration Planning [12], she identified TT needs and proposed a didactic framework to help overcome their unique challenges (See Table 1). For example, TTs are often highly diverse with high task interdependence. They vary in size and dispersion geographically and often struggle with goal misalignment and deep knowledge integration. The transient nature of TT membership further increases the complexity of the translational process [13,14].

**Table 1.** Translational Team (TT) Training Program learning outcomes. Shown are the learning outomes for each session in the first iteration of the TT Training Program

**Session 1** Intro to Team Science	**Session 2** Forming Teams	**Session 3** Launching Teams	**Session 4** Leading Teams	**Session 5** Evaluating Teams
Understand Key Team Science Terms Understand team science benefits and challenges Understand Team Science Best Practices Create a shared mission Cultivate healthy team culture (psychological safety) Facilitate interdisciplinary discussions Build strong research support systems including transparent data management Foster strong leadership Develop an awareness of team science resources	Understand how to facilitate discussions for creation of a shared mission, vision, and goals Understand how to identify team needs to support the shared mission, vision, and goals Understand how to assemble a team of effective collaborators Understand how to cultivate diversity, inclusion, and psychological safety during team development	Understand how to facilitate discussions to generate shared team understanding of approaches, methods, and results Understand how to facilitate discussions to help teams build strong research support systems Understand how to facilitate discussions to ensure team data management is accessible and transparent Understand how to cultivate diversity, inclusion, and psychological safety during different stages of team development	Understand the leadership challenge in translational research Apply principles of transformational leadership to translational research Apply specific leadership strategies to promote Team Science Best Practices	Understand the benefits and challenges of evaluating research teams Articulate the components of an evaluation plan Compare and contrast short- and long-term metrics of success Draft an evaluation plan for an interdisciplinary research team

Rolland et al. describe six evidence-informed best practices from the SciTS essential for overcoming the unique challenges inherent in translational research. The TT Best Practices include: (1) developing a shared mission, vision, and goals, (2) building a culture of trust, accountability, openness, inclusivity, and constant learning, (3) facilitating interdisciplinary conversations on approaches, methods, and results, (4) building robust research support systems, (5) building accessible, transparent data management systems, and (6) fostering strong, functional leadership. Utilized by high-functioning teams, these best practices foster research infrastructures that support reproducible research, promote scientific integrity, and drive good institutional practices [15].

## Design, Build, and Test (the Prototype)

Phase two (Design, build, and test) involves creating a prototype and testing it as part of an iterative process [8]. To that end, we designed, built, and tested a five-session TT Training Program to help translational researchers address their team science challenges.

### Design and Build

Our goal was to create an evidence-informed training program *accessible* for early career researchers; *active* to facilitate learning; and *actionable* so that skills and knowledge could be readily applied after training. To articulate the specific learning outcomes for the TT Training Program, we started with the TT Best Practices as a framework [15]. We then identified learning outcomes as a subset of skills essential for the enactment of the TT Best Practices (See Table 1). For example, the best practice of “Develop a shared mission, vision, and goals,” requires that translational researchers facilitate the development of a mission/vision, adapt the mission/vision as the project evolves, and build consensus to ensure team alignment. The 90-minute sessions were a mix of short lectures and interactive activities, designed to accommodate the busy schedules of translational researchers [4]. Although we designed the five sessions to be part of a cohesive training program, participants were not required to attend them all. Session 1 was conducted in person, but due to a resurgence in COVID-19, the remaining workshops were provided virtually via Zoom. Each session is described briefly below followed by a discussion of the program evaluation plan.

## Session 1: Introduction to Team Science

This session introduced participants to the fundamentals of team science and provided them with knowledge and skills to help their teams function more effectively. The session began by defining team science [13], and the TT Best Practices [15] were shared as practical approaches for overcoming team science challenges.

## Session 2: Forming Breakthrough Science Teams

Session 2 provided participants with knowledge and skills to help them effectively form interdisciplinary teams. Participants then drafted the mission of the research team they would be assembling and discussed how to identify potential team members to best serve that mission. The session concluded by considering issues of diversity and psychological safety when forming a team.

## Session 3: Launching Breakthrough Science Teams

Teams need different skills as they evolve. Therefore, Session 3 provided knowledge and skills for teams after their initial assembly. Participants learned techniques, drawn from the ICTR Team Science Core’s Collaboration Planning intervention, that they could use to facilitate discussions with their teams as they developed their research support systems [12].

## Session 4: Leading Breakthrough Science Teams

We designed Session 4 to strengthen participants' skills and ability to lead interdisciplinary research teams. The session differentiated among leadership models, including transactional, functional, and transformational, and covered the appropriate contexts for each. Finally, participants practiced applying leadership strategies for each of the TT Best Practices [15].

## Session 5: Evaluating Breakthrough Science Teams

This session provided participants with strategies for evaluating their team’s progress and success. Activities for this session centered on creating a draft evaluation plan using a template adapted from materials provided by Sawchuk in a self-guided training for the evaluation of nonprofit organizations [16]. The template included the following components: establishing a shared mission, articulating the project aims, identifying project stakeholders, selecting metrics, and measuring impact.

## TT Training Program Evaluation

Evaluation plans guide the strategic collection of data in order to assess the effectiveness of a training intervention and establish its empirical evidence base [16]. The components of our evaluation plan included: (1) defining the overarching goal of the session(s), (2) identifying the audience, (3) articulating the specific learning outcomes, and (4) describing the theory of change [17]. The overarching goal of our plan was to demonstrate empirically that the training program was accessible, active, and actionable to enhance participants’ abilities as collaborators. The specific audience for the first iteration of the pilot test was postdoctoral fellows at the UW Madison. Learning outcomes for each session were derived in the discover phase (see Table 1).

The next step was to articulate the theory of change expected by the intervention [17]. Although the interactive activities provided participants with an opportunity to engage with content conveyed in the training program, the primary change expected immediately following the sessions was an increase in knowledge and confidence in applying new skills in future collaborations. To measure this change, we evaluated each session using a pretest and posttest design.

The pretest consisted of a short assessment instrument derived from the TT Best Practices [15] and accompanying session learning outcomes (see Supplement, Appendix A). The instrument in Session 1 consisted of 21 statements that began with “I understand how to….” For example, “I understand how to create a culture of trust for my team.” Participants rated each statement on a five-point Likert-type scale ranging from strongly disagree (SD) to strongly agree (SA). The scale was modified for Sessions 2–5 so that respondents indicated their level of knowledge and confidence in their skills on a five-point Likert-type scale that included the following: (1) fundamental awareness, (2) novice, (3) intermediate, (4) advanced, and (5) expert. The metric used during Session 1 assessed respondent knowledge but did not assess actionable skills.

Posttest responses were matched to pretest responses using unique identifiers. The posttests also included additional items to measure the overall value of the sessions. Respondents rated two statements on a five-point Likert-type scale ranging from SD to SA: “The content and skills provided by this workshop will make me a more effective collaborator.” and “I will recommend this workshop to peers and colleagues.” Participants also completed two open-ended questions: “How will you apply what you have learned in this workshop to your collaborative projects?” and “How can we improve the workshop?”

### Test (the Prototype)

To control for the career stage, the audience was limited to postdoctoral fellows at UW Madison (see Supplement, Appendix B). To recruit participants, our team partnered with the Office of Postdoctoral Studies (OPS) at UW Madison. We designed marketing materials with a session summary emphasizing how it could help translational researchers become more effective collaborators. To be as inclusive as possible, we utilized a broad definition of translational research that encompassed all stages from T0 – basic research to T4 – translation to community. The marketing summaries were distributed electronically to all members of ICTR and postdoctoral fellows affiliated with OPS and included on the UW Madison events calendar.

Sessions began in April of 2022 and concluded in November of 2022, with each session held approximately 1.5 months apart. Across all five sessions, there were 52 attendees, with 47 unique attendees, as five participants attended two sessions. Nineteen participants responded to the post-session survey. The average number of participants per session was 10.4 (min = 7, max = 13). Response rates for each session were Session 1: 30%, Session 2: 33%, Session 3: 46%, Session 4: 30%, and Session 5: 43%. Although the overall number of respondents for the session evaluations was low, the results provide helpful information about the utility of the TT Training Program and the necessary steps to take for future improvements.

**Figure 2.**
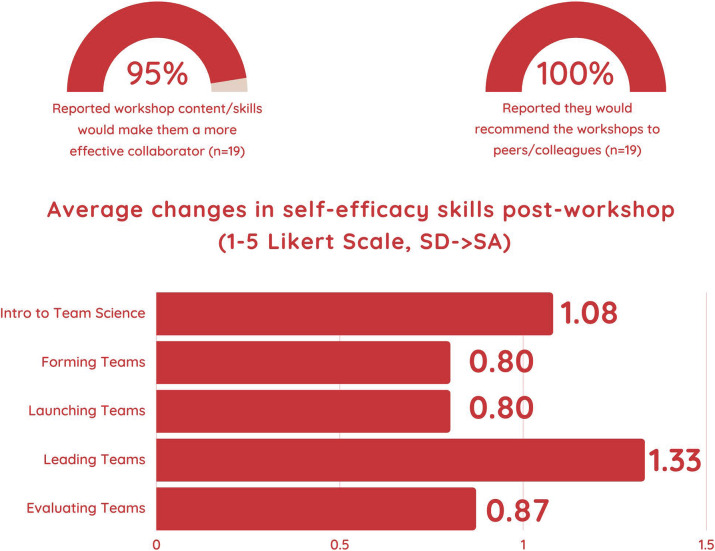
Translational Team (TT) Training Program Quantitative Results. The top portion of this figure shows the percent of respondents who felt the TT Training Program sessions would improve their skills as collaborators and who would recommend the sessions to their colleagues and peers. The total number (n) of respondents is also shown. The bottom portion of the figure shows the average change for the learning outcome measures for each session. All five sessions resulted in an average change of close to one step on the Likert-type scale with Session 4 having the largest change. The Likert-type scale ranged from strongly disagree to strongly agree.

Ninety-five percent of post-session survey respondents indicated that the content and skills provided by the sessions would make them more effective collaborators, and one hundred percent said they would recommend the sessions to their peers and colleagues. Respondents’ scores also increased from pretest to posttest for most of the learning outcomes (see Fig. 2), suggesting that respondent knowledge increased after each session, and they felt greater confidence in applying the learned material (see Supplement, Appendix C). There were a few instances where we saw no change or a decrease from pretest to posttest. After closer review of the individual scores, most of these instances may be explained by the Kruger-Dunning effect, a cognitive bias in which survey respondents who are not familiar with a survey item overestimate their confidence in that item [18]. In these instances, respondents’ initial pretest scores were high. As they became more knowledgeable and more self-aware of the content, they may have responded with less confidence on the posttest. We plan to test this hypothesis in the future by conducting interviews with session participants.

## Revising the TT Training Program

While the preliminary results provided by the pilot test are promising, they also pointed to areas for improvement. Below, we describe the lessons learned and the modifications proposed to improve the overall training experience and create a Revised TT Training Program. While the workshop series was originally based on the TT Best Practices [15], the revised TT Training Program corresponds exactly with the competencies of high-performing TTs as articulated by Brasier *et al*. (2023) [5] (See Table 2).

**Table 2.** Revised TT Training Program learning outcomes. Shown are the session goals and learning outomes for each session in the revised TT Training Program. Each session is mapped onto the appropriate competency domain supporting high-functioning translational teams [5].

**Session 1** Forming Successful Research Teams	**Session 2** Psychological Safety and Team Success	**Session 3** Successful Team Communication and Coordination	**Session 4** Setting Your Science Team Up for (Measuring) Success	**Session 5** Leading Your Team to Success
This session will help you develop knowledge and skills to form effective interdisciplinary research teams for greater innovation and impact.	This session will help you cultivate a psychologically safe culture for your interdisciplinary research collaborations to improve performance and maximize research impact.	This session will help you facilitate interdisciplinary conversations to establish a shared team understanding of processes and systems.	This session will provide skills and resources for creating an effective evaluation plan for interdisciplinary research teams.	This session will help you improve team process and impact with strong transformational leadership.
After completing this session, you will be better prepared to				
Leverage interdisciplinary collaborations to generate more innovative research Engage and motivate prospective collaborators by clarifying your team’s purpose and goals Create maximum research impact by strategically selecting team members and assembling effective expert teams	Understand the impact of psychological safety on team performance in interdisciplinary research teams Assess the psychological safety of an interdisciplinary research collaboration Apply strategies to strengthen team culture by creating a psychologically safe environment	Facilitate discussions to span disciplinary boundaries and develop a shared understanding for an interdisciplinary research collaboration Develop processes for the coordination of an interdisciplinary research collaboration Provide effective feedback and resolve conflicts common to interdisciplinary research collaborations	Select assessment and evaluation methods to monitor performance and impact in interdisciplinary research collaborations Identify and implement metrics that will best demonstrate effectiveness and impact for interdisciplinary research collaborations Create an evaluation plan for a specific interdisciplinary research collaboration to ensure stakeholder satisfaction	Apply evidence-based leadership skills and strategies to improve process and innovation in interdisciplinary collaborations Identify leadership needs for specific interdisciplinary collaborations to achieve more significant scientific impact and innovation Adapt leadership skills and strategies to meet the evolving needs of ongoing interdisciplinary research collaborations
Competency domains supporting high-functioning translational teams [5]				
**Management** Shared Vision Team Member Roles and Responsibilities Project Management	**Affect** Trust Cohesion Psychological Safety	**Team communication** Sharing Knowledge Transactive Memory	**Collaborative problem solving** Learning/Adaptation Collective Intelligence Transdisciplinarity	**Leadership** Sense-making Conflict Resolution Goal Setting

## Changes to Session Delivery

### Explore Real-World Successes

Participants expressed interest in learning more from examples of how actual teams overcame the challenges of collaboration by using the best practices and skills covered in the training program. They specifically mentioned an interest in learning more about “challenges to team science formation” and “using shared mental models to promote collaboration and resolve issues/conflicts.” Our team is working to compile a library of case studies to punctuate training program content. The library will include examples from a variety of disciplines so that workshops can be tailored to different research groups, as the more relatable the material is, the easier it is for participants to absorb and apply to their own work. This change will elevate participant learning along Bloom’s Taxonomy [19], moving from understanding concepts to applying, analyzing, or higher (see Table 2).

### Cultivate Learning Communities

Building a strong sense of community is always a challenge during virtual sessions, and our participants were eager for more peer engagement. As a result, we plan to offer additional ways for participants to interact with each other to create a richer learning experience. The revised training program will intersperse 60-minute Community of Practice Sessions throughout the sessions to provide opportunities to explore personal examples of interdisciplinary collaboration with colleagues.

### Provide Multiple Training Modalities

The first iteration of the training program was designed to enhance the collaborative abilities of individual translational researchers. While it is important for individual researchers to develop their skills, a recent article on TT competencies indicated that team training may be more effective for improving team performance [5]. Therefore, the next iteration of the training program will be offered in multiple modalities. There will be sessions for individual researchers in addition to an option for entire teams. This approach mirrors that of other leading team science training programs (e.g., TeamMAPPS) that provide multiple options for training [20]. Our evaluation instruments will be tailored to these different modalities – individual or team – which will also account for individuals who may not be on a team or may be on multiple teams.

## Changes to Session Outcomes

### Provide Tools for All Team Members

The focus of the first iteration of the training program was on presenting the best practices of team science from the perspective of the team leader. However, we soon discovered that participants also wanted to learn skills they could use as team members on their current teams. The revised TT Training Program includes discussions and activities that allow participants to learn from the experiences of those in roles different from their own. For example, PIs need skills that help them establish and align team members to the mission of the team. This is not a skill that most graduate students or postdocs need while in their current role. Instead, we now also provide skills relevant to more junior team members (e.g., clarifying personal goals, strategically selecting team projects, and aligning their work with the team mission).

### Reduce Time to Application

Session 1 (Intro) piqued participants’ interest in exactly how the TT Best Practices could be used to improve their abilities as collaborators. However, they expressed a desire to “dive deeper” and apply what they learned during this first session. To address this, Session 1 (Intro) and Session 2 (Forming) were combined. The original “Intro” session was more informative and not as active as the other four sessions. Merging it with Session 2 (Forming) allows for the level setting of terms as well as an exploration of actionable strategies and skills in the first session.

### Foster Skills Over Time

This pilot test offered each session as an individual instance with different participants in each session. Although the evaluation results suggest that this mode of presentation was useful, we feel that participants will derive more benefit if they enroll as a complete series. This will not only allow them to build and reinforce their knowledge and skills as they progress through the sequence but it is also the best way for them to improve their skills as interdisciplinary collaborators. The next iteration of the training program will require registration for and participation in all five sessions. To motivate participants to complete all sessions, they will be given the opportunity to earn a digital badge. Digital badges are validated digital records providing reliable, shareable, and verifiable documentation of well-defined and specific knowledge, skills, or competencies. Workshop sessions will be recorded and available for asynchronous viewing for any participants not able to attend all sessions.

## Changes in Session Content

### Increase Content on Psychological Safety

Psychological safety is defined as “…a belief that one will not be punished or humiliated for speaking up with ideas, questions, concerns or mistakes, and that the team is safe for risk-taking [21].” Evaluation respondents explicitly commented that fostering psychological safety was an important concern. In the first iteration of the training program, team climate and psychological safety were limited to subsections 1, 2, and 3. The revised training program now has a session focused specifically on this topic. This is in line with a recent paper indicating that psychological safety and trust are essential for high-functioning TTs [5].

### Increased Emphasis on Communication for Better Team Coordination

Session 3, Launching Breakthrough Science Teams, provided knowledge and skills for teams after their initial assembly. Participants learned techniques they could use to facilitate discussions with their teams as they developed their research support systems [12] and policies. In addition, this session also covered the importance of establishing psychological safety when launching a team.

Because the bulk of the material covered in Session 3 was focused on the communication needed for new collaborations, it was renamed as “Successful Team Communication and Coordination.” Effective knowledge sharing is one of the three most important competencies for high-functioning TTs, and, thus, it is an appropriate focus for future iterations of the training intervention [5]. Consequently, a new section was added that addressed giving and receiving effective feedback, and the material about psychological safety was moved to Session 2.

### Limitations

The audience for this pilot test was limited to postdoctoral fellows at the UW Madison. As a result, there may be some limits to the generalizability of our findings. To ensure the revised TT Training Program is effective for early career faculty and scientists, we will need to expand our audience and align content so that it is maximally relevant to other career stages. To do this, we will need to collect additional information from our potential participants during the registration process. For example, it would be helpful to know more about their experience in working with different types of research teams, including translational teams. We are currently pilot-testing the revised version with a broader audience from the CAIRIBU Consortium (Collaborating for the Advancement of Interdisciplinary Research in Benign Urology). As part of this second pilot test, we collected additional information about participants’ team experience to better tailor our efforts. In addition, we will be measuring the retention of learning by conducting a six-month follow-up evaluation.

As we expand our audience, we also hope to improve our evaluation response rate. While the results provide initial evidence that the workshops enhanced the collaborative abilities of translational researchers, the average response rate for posttest survey completion was only 36.4%. To effectively establish a strong empirical evidence base, additional data will need to be collected. We also need to acknowledge that measures of self-report can often be biased. Future iterations will involve additional assessment methods (e.g., interviews and focus groups) to further strengthen program evaluations

## Conclusion

Articulating the KSAs and building the evidence base for effective team science training will make it easier for research institutes like CTSA hubs to strengthen the collaborative abilities of translational researchers and enhance their ability to impact human health [5,8]. To that end, we created a five-session TT Training Program using best practices from the SciTS as a framework [15]. Evaluation respondents indicated that the sessions would improve their skills as collaborators and they would recommend the sessions to peers and colleagues. Respondent skill level improved for most skills measured, but response rates were low and additional data need to be collected for conclusive results.

Evaluation responses from this first pilot study also suggested ways to improve the next iteration of the training program. The revised TT Training Program will incorporate ways to strengthen evaluation, provide quicker access to skills for all team members, incorporate more material about team climate and psychological safety, engage actively with real-world examples in peer-based learning communities, and cement learning over time by requiring participation in all sessions. These changes will complement the framework provided by the TT Best Practices and ultimately guide translational researchers toward richer collaborations with greater impact [15].

## Supporting information

Sweeney et al. supplementary material Sweeney et al. supplementary material

## Supplementary material

The supplementary material for this article can be found at https://doi.org/10.1017/cts.2023.649.
