# Supplementary material for: Developing a Translational Team Training Program using the Wisconsin Interventions in Team Science Framework
Source: J Clin Transl Sci. 2023 Oct 19;7(1):e233. doi: 10.1017/cts.2023.649 (PMC10643908; doi:10.1017/cts.2023.649)
Supplement: Sweeney et al. supplementary material [file S2059866123006490sup001.docx]

**Supplemental Information**

**Appendix A: TT Training Program Evaluation Methodology**

**Session 1 (In Person)**

The first session was planned and conducted as an in-person event. Interested participants completed a registration form that asked for their name, email, school/college/center, and academic department. Participants were also asked if they would like to receive information about future team science related events. Surveys for Session 1 were completed using Qualtrics. Evaluation was performed in accordance with IRB 2017-0860-CP007 and participants opted in by completing the evaluations. Session 1 included three surveys: A registration form, a pre-test, and a post test. Questions for each survey are presented below. Twenty-nine individuals completed the initial registration form (See Appendix B). All but one participant indicated that they would like additional information about upcoming team science events.

A few days prior to the session, registrants were contacted and asked to complete a pre-test. The pre-test first asked about the stage of team development. Answers started with “working as a co-investigator on a project” which is appropriate for those currently collaborating on others’ projects. Additional answers moved through, “applying for a pilot award,” “launching a pilot project,” “applying for larger federal funding,” culminating with “launching a large project, center or institute.” The next item asked about leadership status (e.g., co-investigator, leader of an independent lab, leader of a multi-investigator project, or leader of a large collaboration). The survey for the first session also asked about whether registrants were conducting translational research.

The pre-test then asked each respondent to rate on a Likert-type scale (strongly disagree to strongly agree) their level agreement with statements of their understanding of team science knowledge and skills. Immediately following the session, participants were sent a post-test with the same Likert-type items as the pre-test. Participants were also asked to indicate if the session content and skills would make them an effective collaborator and if they would recommend the session to the colleagues and peers. Finally, participants were asked to provide information to help improve the training program.

**Session 1: Registration Form Items**

This workshop is currently open to post-doctoral researchers at the University of Wisconsin. Please indicate if you meet these criteria (yes or no). If you do not meet these criteria but would like information about future Team Science events, please fill out the rest of the form and check “Yes” to be added to our email list.

Please provide the following information:

- First name
- Last name
- Email
- School, College, or Center
- Academic Department

We will soon be rolling out additional Team Science courses and workshops for researchers and staff at all career stages. Are you interested in receiving information about future Team Science events? (yes or no)

**Session 1: Pre-test Items**

Please indicate which stage of team development best fits your current research engagement:

- Working as a co-investigator on a project
- Applying for a pilot award
- Launching a pilot project
- Applying for larger federal funding
- Launching a large project, center, or institute

Please indicate which statement best describes your current leadership status.

- I am a co-investigator on a collaborative project
- I lead an independent lab
- I lead a multi-investigator project
- I lead a large collaboration, center, or institute

Translational Teams generate new knowledge with the goal of advancing an interventional product or evidence-based approach to improve human health. Would you classify your research collaboration(s) as translational research? (yes or no)

If yes, please select the stage that best defines your translational team’s current research:

- T0 Basic Science Research (Preclinical and animal studies): Defining mechanisms, targets and lead molecules
- T1 Translation to Humans (Proof of concept, Phase 1 clinical trials): New methods of diagnosis, treatment, and prevention
- T2 Translation to Patients (Phase II or Phase III clinical trials): Controlled studies leading to effective care
- T3 Translation to Practice (Phase IV clinical trials and clinical outcomes research): Delivery of recommended and timely care to the right patient
- T4 Translation to Community (Population-level outcomes research): Broad benefit to society

Please rate your agreement with the following phrases using the following scale (Strongly Disagree, Disagree, Neutral, Agree, Strongly Agree).

- I understand how to help my team develop a research mission and vision
- I understand how to help my team establish goals and metrics for success.
- I understand how to help my team members clearly define their roles and responsibilities.
- I understand how to create a culture of trust for my team.
- I understand how to create a culture in which my team members are held accountable.
- I understand how to create a culture of openness for my team.
- I understand how to create a culture of inclusivity for my team.
- I understand how to create a team culture in which constant learning is valued.
- I understand how to create a team culture that is psychologically safe.
- I understand how to facilitate interdisciplinary conversations about theoretical approaches.
- I understand how to facilitate interdisciplinary conversations about different research methods.
- I understand how to facilitate interdisciplinary conversations about different types of data review and analysis.
- I understand how to facilitate interdisciplinary conversations about different ways to interpret results.
- I understand how to help my team develop an information management system to support team functioning.
- I understand how to help my team develop a scientific coordination system and project management plan to fulfill team objectives.
- I understand how to help my team develop a communication plan to make information clear and accessible.
- I understand how to help my team develop a data management system that is accessible and transparent.
- I understand how to mentor and coach to my team members.
- I understand how to help my team monitor its performance.
- I understand how to provide my team with useful feedback.
- I understand how to help my team build capacity.

What are your goals for participating in this project?

**Session 1: Post-test Items**

Please rate your agreement with the following phrases using the following scale (Strongly Disagree, Disagree, Neutral, Agree, Strongly Agree).

- I understand how to help my team develop a research mission and vision.
- I understand how to help my team establish goals and metrics for success.
- I understand how to help my team members clearly define their roles and responsibilities.
- I understand how to create a culture of trust for my team.
- I understand how to create a culture in which my team members are held accountable.
- I understand how to create a culture of openness for my team.
- I understand how to create a culture of inclusivity for my team.
- I understand how to create a team culture in which constant learning is valued.
- I understand how to create a team culture that is psychologically safe.
- I understand how to facilitate interdisciplinary conversations about theoretical approaches.
- I understand how to facilitate interdisciplinary conversations about different research methods.
- I understand how to facilitate interdisciplinary conversations about different types of data review and analysis.
- I understand how to facilitate interdisciplinary conversations about different ways to interpret results.
- I understand how to help my team develop an information management system to support team functioning.
- I understand how to help my team develop a scientific coordination system and project management plan to fulfill team objectives.
- I understand how to help my team develop a communication plan to make information clear and accessible.
- I understand how to help my team develop a data management system that is accessible and transparent.
- I understand how to mentor and coach to my team members.
- I understand how to help my team monitor its performance.
- I understand how to provide my team with useful feedback.
- I understand how to help my team build capacity.

The content and skills provided by this workshop will make me a more effective collaborator (Strongly Disagree, Disagree, Neutral, Agree, Strongly Agree).

I will recommend this workshop to my peers and colleagues (Strongly Disagree, Disagree, Neutral, Agree, Strongly Agree).

How will you apply what you learned in this workshop to your collaborative projects?

How can we improve the workshop?

**Sessions 2-5 (Virtual)**

Due to a resurgence in COVID, we decided to conduct the remaining sessions virtually via Zoom. At this time, we also transitioned from using Qualtrics to conduct the assessments to REDCap to help unify the evaluation process within our CTSA. To further streamline our process, we decided to combine the registration form and pre-test. The idea was that reducing the number of surveys to complete would promote participation in the evaluation process. On average, response rates were 8% higher for sessions 2-5 (see Appendix C). The items for the pre- and post-tests for Sessions 2-5 are shown below.

**Sessions 2-5: Registration and Pre-test Items**

**Common Items**

This workshop is currently open to post-doctoral researchers at the University of Wisconsin. Please indicate if you meet these criteria (yes or no). If you do not meet these criteria but would like information about future Team Science events, please fill out the rest of the form and check “Yes” to be added to our email list.

Please provide the following information:

- First name
- Middle Name/Initial
- Last name
- Email
- Institutional Affiliation

We will soon be rolling out additional Team Science courses and workshops for researchers and staff at all career stages. Are you interested in receiving information about future Team Science events? (yes or no)

**Demographic Questions**

This set of demographic questions is standardly used for evaluations conducted by the UW Madison Institute for Clinical and Translational Research. Because of the small sample size with this project, we did not conduct any analyses based on demographic information.

Are you of Hispanic or Latino origin? (yes or no)

What Hispanic/Latino origin do you identify with (check all that apply)?

- Cuban
- Puerto Rican
- Mexican/Mexican-American
- Chicano/Other Hispanic-Latino

With which racial or ethnic backgrounds to you identify?

- American Indian or Alaska Native
- Native Hawaiian or Other Pacific Islander
- Black or African American
- White
- Cambodian
- Hmong
- Laotian
- Vietnamese
- Other Asian

Which of these descriptors best represents your gender identity?

- Female
- Male
- Nonbinary/gender nonconforming
- Identity not listed
- Prefer not to answer

**Affiliations**

School or College Primary Appointment (list provided)

Academic Department

**Session 2 Learning Outcomes**

How do you rate your knowledge of or skills in the following areas from (1) fundamental awareness to (5) expert?

- Developing a research mission.
- Selecting team members that meet the needs of the team.
- Defining team member roles and expectations.
- Creating a culture of inclusivity for my team.
- Creating a team culture that is psychologically safe.

**Session 3 Learning Outcomes**

How do you rate your knowledge of or skills in the following areas from (1) fundamental awareness to (5) expert?

- Facilitating interdisciplinary conversations to generate shared mental models.
- Creating a culture of inclusivity and psychological safety.
- Selecting research support systems that will benefit the team.
- Leveraging available resources for support system development.
- Ensuring my team data management system is accessible and transparent.

**Session 4: Learning Outcomes**

How do you rate your knowledge of or skills in the following areas from (1) fundamental awareness to (5) expert?

- Understanding the leadership challenge in translational research.
- Applying the principles of different leadership models to translational research.
- Employing specific leadership strategies to promote team science best practices.

**Session 5: Learning Outcomes**

How do you rate your knowledge of or skills in the following areas from (1) fundamental awareness to (5) expert?

- Understanding the benefits of evaluating a research team.
- Understanding the challenges of evaluating a research team.
- Comparing and contrasting metrics for measuring short-term progress and long-term impact.
- Defining the components of an evaluation plan for teams.
- Creating an evaluation plan for your research team.

**Sessions 2-5: Post-test Items**

**Session 2-5 Learning Objectives (see above)**

**Common Items**

The content and skills provided by this workshop will make me a more effective collaborator (Strongly Disagree, Disagree, Neutral, Agree, Strongly Agree).

I will recommend this workshop to my peers and colleagues (Strongly Disagree, Disagree, Neutral, Agree, Strongly Agree).

How will you apply what you learned in this workshop to your collaborative projects?

How can we improve the workshop?

**Appendix B: TT Training Program Pilot Participants**

| **Description of Participants and Number of Surveys Completed** | | | | | | |
| --- | --- | --- | --- | --- | --- | --- |
| **Session** | **Departments Represented by Registrants** | **Completed Pre-tests** | **Session**  **Participants** | **Completed Post-tests** | **Race/Ethnicity** | **Gender Identity** |
| 1  (Intro) | Agricultural Economics, Agronomy, Bacteriology, Cardiovascular Medicine, Chemistry, Computer Science, Engineering,  Family Medicine and Community Health, Forest and Wildlife Ecology, Geoscience, Medical Physics, Nutritional Sciences, Oncology, Pathology, Plant Pathology, Psychiatry, Radiology, Soil Science, Surgery | 8* | 10 | 3 | Not available | Not available |
| 2  (Forming) | Aquatic Sciences, Bacteriology, Biostatistics and Medical Informatics, Chemistry, Family Medicine and Community Health, Geography, Oncology, Ophthalmology and Visual Sciences, Radiology, Surgery | 31 | 12 | 4 | 25% Other Asian  58% White  17% Unknown | 58% Female  33% Male  9% Unknown |
| 3  (Launching) | Biochemistry, Quantitative Cell Imaging, Computer Science, Engineering, Endocrine and Reproductive Health, Environmental Studies, Family Medicine and Community Health, Humanities, Medical Microbiology and Immunology, Obstetrics and Gynecology | 23 | 13 | 6 | 23% Other Asian  8% Hispanic/Latino (Mexican/Mexican American)  69% White | 69% Female  31% Male |
| 4  (Leading) | Animal and Dairy Sciences, Biomolecular Chemistry, Demography of Health and Aging, Engineering, Genetics, Research on Poverty, Integrative Biology, Primate Center | 18 | 10 | 3 | 30% Other Asian  10% Hispanic/Latino (Chicano)  20% Black/ African  American  30% White  10% Unknown | 60% Female  40% Male |
| 5  (Evaluating) | Bacteriology, Human Development and Family Services, Medical Microbiology and Immunology, Plant Pathology | 13 | 7 | 3 | 29% Other Asian  14% Hispanic/Latino (Chicano)  14% Black/African  American  43% White | 29% Female  71% Male |
| Totals |  | 93 | 52** | 19 |  |  |
| Means |  | 18.6 | 10.4*** | 3.8 |  |  |
| *Session 1 had a separate registration form. Twenty-nine individuals completed the registration form. Only 8 individuals completed the pre-test.  **There were 47 unique participants. Five of the 52 total participants completed two of the training program sessions.  ***Based on total number of participants (n=52) | | | | | | |

**Appendix C: TT Training Program Average Difference Scores for Each Learning Outcome**

| **Average Difference Scores for Session 1** | |
| --- | --- |
| **Training Program Assessment Tool Items** | **Average Difference Scores**  **n=3/10,**  **30% response rate** |
| I understand how to help my team develop a research mission and vision | 1.67 |
| I understand how to help my team establish goals and metrics for success. | 0.67 |
| I understand how to help my team members clearly define their roles and responsibilities. | 1.67 |
| Shared Mission, Vision, and Goals (average) | 1.33 |
| I understand how to create a culture of trust for my team. | 0.00 |
| I understand how to create a culture in which my team members are held accountable. | 0.67 |
| I understand how to create a culture of openness for my team. | 1.33 |
| I understand how to create a culture of inclusivity for my team. | -0.33 |
| I understand how to create a team culture in which constant learning is valued. | 1.33 |
| I understand how to create a team culture that is psychologically safe. | 1.33 |
| Team Culture (average) | 0.72 |
| I understand how to facilitate interdisciplinary conversations about theoretical approaches. | 1.00 |
| I understand how to facilitate interdisciplinary conversations about different research methods. | 2.00 |
| I understand how to facilitate interdisciplinary conversations about different types of data review and analysis. | 1.00 |
| I understand how to facilitate interdisciplinary conversations about different ways to interpret results. | 0.67 |
| Interdisciplinary Conversations (average) | 1.17 |
| I understand how to help my team develop an information management system to support team functioning. | 1.33 |
| I understand how to help my team develop a scientific coordination system and project management plan to fulfill team objectives. | 2.00 |
| I understand how to help my team develop a communication plan to make information clear and accessible. | 1.33 |
| I understand how to help my team develop a data management system that is accessible and transparent. | 2.00 |
| Research Support Systems (average) | 1.67 |
| I understand how to mentor and coach to my team members. | 0.67 |
| I understand how to help my team monitor its performance. | 1.00 |
| I understand how to provide my team with useful feedback. | -0.33 |
| I understand how to help my team build capacity. | 0.67 |
| Leadership (average) | 0.50 |
| Participants rated each statement on a five-point Likert-type scale ranging from Strongly Disagree to Strongly Agree. | |

| **Average Difference Scores for Sessions 2-5** | |
| --- | --- |
| **Session 2: Forming Teams** | n=4/12  33% response rate |
| Knowledge/skills in developing a research mission | 0.60 |
| Knowledge/skills selecting team members that meet the needs of the team | 0.80 |
| Knowledge/skills defining team member roles and expectations | 0.80 |
| Knowledge/skills creating a culture of inclusivity for my team | 1.00 |
| Knowledge/skills creating a team culture that is psychologically safe | 0.80 |
| **Session 3: Launching Teams** | n=6/13  46% response rate |
| Facilitating interdisciplinary conversations to generate shared mental models | 0.00 |
| Creating a culture of inclusivity and psychological safety | 0.00 |
| Selecting research support systems that will benefit the team | 1.00 |
| Leveraging available resources for support system development | 1.00 |
| Ensuring my team data management system is accessible and transparent | 2.00 |
| **Session 4: Leading Teams** | n=3/10  30% response rate |
| Understanding the leadership challenge in translational research | 1.00 |
| Applying the principles of different leadership models to translational research | 1.33 |
| Employing specific leadership strategies to promote team science best practices | 1.67 |
| **Session 5: Evaluating Teams** | n=3/7  43% response rate |
| Understanding the benefits of evaluating a research team | 0.67 |
| Understanding the challenges of evaluating a research team | 1.00 |
| Comparing and contrasting metrics for measuring short-term progress and long-term impact | 0.67 |
| Defining the components of an evaluation plan for teams | 1.00 |
| Creating an evaluation plan for your research team | 1.00 |
| Participants rated their knowledge and skills on a five-point Likert-type scale that included the following data points (1) fundamental awareness, (2) novice, (3) intermediate, (4) advanced, and (5) expert | |

**Appendix D: Qualitative Responses to “How will you apply what you have learned in this workshop?**

**Session 1 (Intro) respondents plan to**

- Create a safe and balanced working environment where everyone’s contribution is valued.
- Create a welcoming and inclusive environment.
- Create an environment where team members can openly share concerns.

**Session 2 (Forming) respondents plan to**

- Identify a shared team mission that will guide team formation and interactions.
- Create a psychologically safe environment.
- Engage in open discussions and appreciate team efforts to boost confidence
- Review team progress and align with best practices.

**Session 3 (Launching) respondents plan to**

- Establish a common goal.
- Identify metrics to assess progress and success.
- Determine how responsibilities and credits should be shared.
- Strategically plan meetings.
- Address conflict right away.
- Guide team towards a shared mental model.

**Session 4 (Leading) respondents plan to**

- Learn more about psychological safety.
- Codify a shared vision and revisit long-term goals.
- Clarify roles.

**Session 5 (Evaluating) respondents plan to**

- Initiate discussions about team goals and push for more specific aims.
- Strategically select team members.
